# Supplementary material for: The interactome of intact mitochondria by cross-linking mass spectrometry provides evidence for coexisting respiratory supercomplexes
Source: Mol Cell Proteomics. 2017 Dec 8;17(2):216–32. doi: 10.1074/mcp.RA117.000470 (PMC5795388; doi:10.1074/mcp.RA117.000470)
Supplement: Supplemental Data [file supp_RA117.000470_133922_0_supp_23978_fzffwf.zip › spectra_annotation/mito_DR_spectra_annotation/333-1-1-1-7-1.pdf]

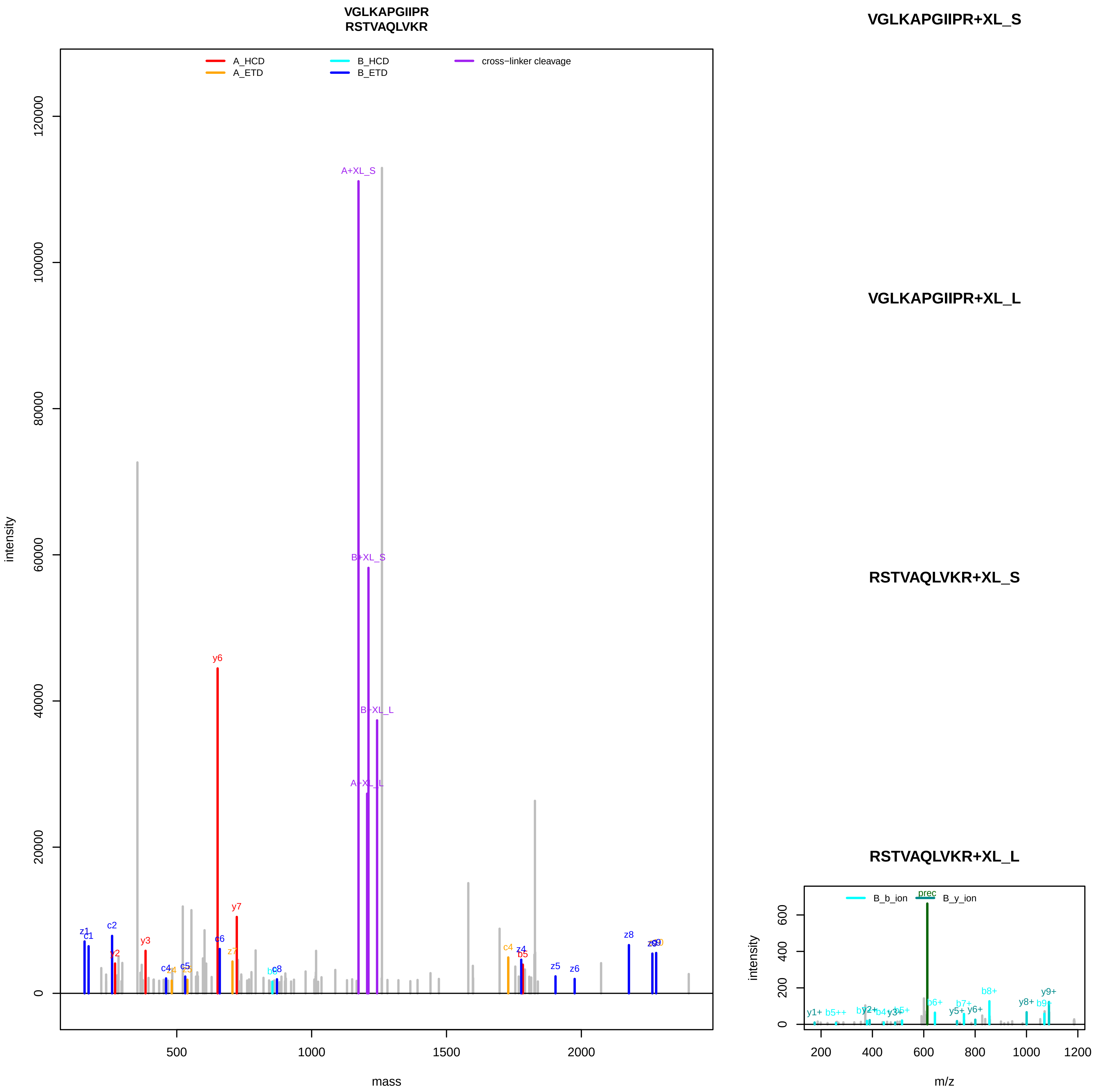

VGLKAPGIIPR
RSTVAQLVKR
A_HCD
A_ETD
B_HCD
B_ETD
cross−linker cleavage
A+XL_S
B+XL_S
B+XL_L
A+XL_L
y6
y7
y3
y2
z1
c1
c2
c4
c5
c6
c8
c4
z4
b5
z5
z6
z8
z9
c9
intensity
mass
0
20000
40000
60000
80000
100000
120000
500
1000
1500
2000
VGLKAPGIIPR+XL_S
VGLKAPGIIPR+XL_L
RSTVAQLVKR+XL_S
RSTVAQLVKR+XL_L
B_b_ion
B_y_ion
prec
y1+
b5++
y2+
y3+
b6+
b7+
y5+
y6+
b8+
y8+
y9+
intensity
m/z
0
200
400
600
200
400
600
800
1000
1200
